# Investigating the early uptake of digital self-management interventions amongst Dutch cancer survivors using nationwide registry data—the OncoAppstore case

**DOI:** 10.1007/s00520-025-10035-5

**Published:** 2025-10-17

**Authors:** L. van Deursen, A. M. de Korte, R. van der Vaart, J. J. Aardoom, M. M. Stouten, C. R. M. Lammens, N. H. Chavannes, J. N. Struijs

**Affiliations:** 1 Department of Population Health and Health services research, Center for Public health, Healthcare and Society, National Institute for Public Health and the Environment, Antonie Van Leeuwenhoeklaan 9, 3721 MA Bilthoven, The Netherlands; 2 National eHealth Living Lab, Leiden, the Netherlands; 3 Netherlands Comprehensive Cancer Organization, Utrecht, The Netherlands; 4 CoRPS - Centre of Research On Psychological Disorders and Somatic Diseases, Department of Medical and Clinical Psychology, Tilburg University, Tilburg, The Netherlands; 5 Research Group Technology for Healthcare, The Hague University of Applied Sciences, The Hague, The Netherlands; 6 Department of Public Health and Primary Care, Leiden University Medical Center, Leiden, The Netherlands; 7 Health Campus The Hague, Department of Public Health and Primary Care, Leiden University Medical Center, Turfmarkt 99, The Hague, The Netherlands; 8 Dutch Healthcare Authority, Utrecht, the Netherlands; 9 National AYA ‘Young and Cancer’ Network, Amsterdam, The Netherlands

**Keywords:** E-health, Cancer, Self-management, Uptake, Health disparities, Survivorship

## Abstract

**Purpose:**

Digital self-management interventions can aid cancer survivors with psychosocial, emotional, physical, and lifestyle support needs, but usage is relatively low due to a lack of awareness and reimbursement. Additionally, there are concerns about e-health exacerbating existing health disparities. To enhance the uptake of digital self-management interventions, the OncoAppstore was launched—a landing page on a well-known Dutch platform that offers cancer survivors credits to purchase these interventions. It is unknown to what extent the nature of the OncoAppstore will influence uptake and appeal to a diverse group of cancer survivors. The current study therefore aimed to explore sociodemographic, clinical, income, and healthcare usage factors associated with its early uptake among survivors.

**Methods:**

OncoAppstore usage data were combined with nationwide registry data from Statistics Netherlands, the Netherlands Cancer Registry, and Dutch Hospital Data. Data were analysed descriptively, and differences between OncoAppstore users and non-users were statistically tested using chi-square tests, Fisher’s exact tests, and Mann–Whitney *U* tests.

**Results:**

OncoAppstore users tended to have higher educational levels and incomes and were more likely to be female, younger on average, and more often of Dutch origin compared to non-users. Users had higher primary and specialist mental healthcare expenditures and lower expenditures on total healthcare, general practitioner, hospital, and pharmaceutical care.

**Conclusion:**

The early users of the OncoAppstore appear to be a selective group of cancer survivors as they are primarily individuals with high socioeconomic status. Thus, consistent with other e-health research, the same user patterns are observed. Future research should investigate whether this trend persists after a longer period and what is needed to better reach underrepresented groups.

## Introduction

As cancer survival rates continue to rise, the number of individuals living with the long-term effects of cancer disease and treatment, such as fatigue, fear, depression, cognitive issues, and neuropathy, is also increasing [1, 2]. Consequently, there is a growing need for psychosocial, emotional, physical, and lifestyle support. While some support is available, it is often constrained by factors like specialist availability, financial resources, and facilities. Previous studies have shown that a large group of cancer survivors (i.e. those living with cancer from the time of diagnosis until the end of their life [3]) do not receive the needed support and have unmet healthcare needs [4–6].

An efficient and effective manner to provide psychosocial or lifestyle support and to help survivors with their (disease and treatment-related) needs is through digital self-management interventions. Many effective digital self-management interventions have been developed, such as interventions to monitor health-related quality of life or provide psychosocial, physical, emotional, and lifestyle support for cancer survivors [7–10]. Digital self-management interventions have been proven effective in improving a range of health outcomes, including symptom burden, pain complaints, lifestyle, psychological adjustment, and overall quality of life, as well as reducing side effects of the disease and treatment such as cancer-related anxiety, fatigue, or depressive symptoms [5, 7, 11–13]. Despite the potential benefits of digital self-management interventions, multiple challenges to their uptake exist. First, many cancer survivors are unaware of their existence and proven benefits. Second, there is a general reluctance among cancer survivors to invest in these digital tools, as they are generally unwilling or unable to pay for programs that come with a cost [14, 15]. Third, reimbursement remains a barrier: in the Netherlands, there is currently no structural funding mechanism, and consequently, no reimbursement schemes exist for platforms such as the OncoAppstore [14, 15]. Finally, some survivors perceive them as being inferior to in-person support from healthcare providers [15]. Likely influenced by these challenges, only a small number of cancer survivors have used digital self-management interventions [15].

Furthermore, there are large differences in e-health usages among different groups of users. For example, women are more likely to search for health information online and to use mobile applications [16–27]. Other research suggests that higher age, lower income, lower education, living alone, and residing in rural areas (i.e. with less healthcare in proximity) are associated with lower e-health use, while findings on e-health usage among racial and ethnic groups remain mixed [16–25, 28–32]. Multiple scholars expressed their concerns about digital interventions exacerbating health disparities, primarily benefiting individuals with more resources [16–23, 29].

In July 2023, the OncoAppstore was launched in the Netherlands to enhance the uptake of digital self-management interventions among cancer survivors and their relatives. The OncoAppstore is a landing page on ‘Kanker.nl’ (in English: Cancer.nl)—a well-known platform in the Netherlands—and serves as a central hub that provides evidence-based digital self-management interventions [33]. The platform’s primary aim is to enhance and simplify access to evidence-based self-management interventions for cancer survivors [34]. Users of the OncoAppstore are provided with a temporary personal digital health credit of 100 euros, eliminating financial barriers. This credit allows them to access a curated selection of high-quality digital interventions. Potential users are informed about the credit on the platform.

It is unknown to what extent the nature of the OncoAppstore—being offered on a well-known platform with a selection of high-quality interventions and providing a digital health credit—will influence uptake among cancer survivors and appeal to a diverse group of survivors. It could be the case, for example, that individuals with lower income will make more use of digital self-management interventions once the financial barrier is removed or that females will use it more as they are more likely to search for health information online and to use apps [16–27]. Therefore, this exploratory study aims to examine the early uptake of the OncoAppstore and to explore sociodemographic, clinical, income, and healthcare usage factors associated with its uptake among cancer survivors, by comparing users of the OncoAppstore with the general population of cancer survivors (non-users).

## Method

### Study setting

The OncoAppstore is an initiative of three Dutch cancer-related organizations: The Netherlands Comprehensive Cancer Organization (Dutch abbreviation: IKNL), the online platform ‘Kanker.nl’, and the Cancer Society (Dutch abbreviation: KWF) [35]. IKNL is the quality institute for oncological and palliative research and practice in the Netherlands [36]. ‘Kanker.nl’ is a well-known platform among cancer survivors and healthcare professionals with over 1,000,000 visits per month [37]. It provides information and support and is a forum for cancer survivors and their relatives [38]. KWF is an organization that aims to facilitate cancer research, influence policy, and share knowledge [39]. KWF temporarily provides the digital health credit to OncoAppstore users; a doctor or practitioner referral is not required. Those interested in using the OncoAppstore must create a personal account on Kanker.nl. Once registered, they can claim the digital health credit to purchase interventions [40].

IKNL, Kanker.nl, and the Dutch Public Health Service (Dutch abbreviation: GGD GHOR) have tested all interventions offered in the OncoAppstore for usability, reliability, scientific foundation, and privacy protection. The testing was carried out using the GGD test method for digital applications [41]. The OncoAppstore offers various interventions to support individuals with challenges caused by the disease and its treatment. These include a decision-making aid for those considering (additional) treatment options, mental health support for those dealing with issues such as depression, low self-esteem, and fear of recurrence, as well as interventions to help improve physical health [33].

KWF, IKNL, Kanker.nl, and the organizations responsible for the interventions promote the OncoAppstore through social media campaigns and digital newsletters. Information is available on the websites of several cancer patient organizations and hospitals, and promotional material is being distributed via the waiting rooms of general practitioners in several municipalities within the Netherlands. This contributes to the awareness of digital self-management interventions among cancer survivors. Furthermore, presentations and other events for healthcare professionals are being held.

### Study design, data sources, and study measures

This study is part of the broader OncoAppstore research, which investigates the OncoAppstore and consists of multiple studies. The methods and related studies can be found in a protocol paper [42]. The study was submitted and exempt from review by the Medical Research Ethics Committee Brabant (NW2022-42), as this study is not subject to the Dutch Medical Research Involving Human Subject Act. The current exploratory observational study combines data from three different sources into a single dataset.

The first data source is IKNL. Individuals interested in using the digital health credit on the national online cancer platform Kanker.nl were invited to voluntarily participate in scientific research. Those who agreed received an information letter and informed consent form from IKNL. By digitally signing the consent form, participants also consented to their data being linked with data from Statistics Netherlands (SN, Dutch abbreviation: CBS) and the Netherlands Cancer Registry (NCR, Dutch abbreviation: NKR) in compliance with the European General Data Protection Regulation [43]. IKNL provided information about which cancer survivors have used the OncoAppstore (i.e. those who have purchased at least one intervention and provided informed consent) between July 2023 and October 2024. Data on cancer diagnosis and year of diagnosis for OncoAppstore users were obtained from the NCR, which is managed by IKNL [44–46].

The second data source was the National Basic Hospital Care Register data obtained from Dutch Hospital Data (DHD), which contains information on cancer diagnoses. DHD is an organization that collects, manages, and edits hospital data [46, 47]. The National Basic Hospital Care Register contains information on hospital admissions and associated diagnoses in Dutch hospitals of individuals registered in the Dutch Personal Records Database based on ICD10 codes [46]. The study used Dutch Hospital Data from 2018 to 2022 (the most recent year of which data were available) to include all individuals admitted to the hospital at least once with a cancer diagnosis (ICD10 codes C00–C97, excluding C44) [44] to be able to compare OncoAppstore users with the general population of cancer survivors (non-users). The year of diagnosis was determined based on the first time individuals were admitted to the hospital for a cancer diagnosis. The year of diagnosis and type of non-users diagnosed before 2018 or after 2022 could not be determined as no data were available. Individuals who were deceased before the launch of the OncoAppstore (July 2023) were filtered out and excluded from the analyses.

The third data source was the System of Social Statistical Datasets from Statistics Netherlands [48, 49], which provides data on socio-demographics, income, healthcare usage, and care proximity. The System of Social Statistical Datasets is a standardized system of interlinked registers and surveys that contains information on persons, households, jobs, benefits, education, hospitalizations, and more [49]. Researchers can access these data via remote access, granted under strict privacy-securing conditions [49].

The OncoAppstore usage data of IKNL, the NCR data, and the DHD were uploaded to the remote access environment of Statistics Netherlands and linked to the Statistics Netherlands data. Statistics Netherlands replaced all personal identifiers from all data sources with an anonymous key, called a PIN (personal identification number), to link the data before sharing it with researchers, in order to protect the privacy of individuals and prevent direct identification [49]. Also, the output of the researchers is checked by Statistics Netherlands to ensure the privacy and anonymity of participants.

The variables used from Statistics Netherlands are described below.

#### Sociodemographic and income-related characteristics

The sociodemographic and income-related data included gender (male or female), age (in years), level of education, country of origin, income, benefits, and debts. These variables were included in the analysis because differences between OncoAppstore users and non-users were expected based on these characteristics. Research has shown that individuals with a low socioeconomic status, a migrant background, or older age often experience greater difficulties in independently searching for, accessing, understanding, and utilizing e-health interventions. Also, women are more likely to search for health information online and to use mobile applications [16–27]. Results on the influence of ethnicity are mixed [28, 32].

Education level was classified as high, middle, and low, according to Statistics Netherlands’s categorization [50]. Country of origin was categorized into three groups: (1) Netherlands, (2) Europe (excluding the Netherlands), and (3) Outside of Europe [51]. The income of individuals was based on standardized household income compared with other households, excluding those of students. The study classified household incomes into three income classes: low income (percentile 0 to 40), middle income (percentile 40 to 80), and high income (percentile 80 to 100) based on the classification of Statistics Netherlands. Also, an analysis was conducted to determine whether individuals aged 66 years or younger received benefits (i.e. unemployment benefits, disability benefits, social assistance benefits, or other social security benefits). Only individuals under the age of 67 were included, as everyone in the Netherlands receives public pension benefits starting at the age of 67. Individual debts were assessed by checking whether individuals were in debt restructuring, were health insurance defaulters, or both (as a proxy for financial distress). Individuals who received a debt rescheduling order under the Natural Persons Debt Rescheduling Act 34 were considered to be in debt restructuring [52]. Health insurance defaulters were adults registered in the Personal Records Database who were obliged to take out insurance under the Healthcare Insurance Act but had not paid a premium for their primary insurance for at least 6 months and were subject to the administrative law premium regime.

#### Healthcare expenditures and reception of additional care

In the analysis, data on healthcare expenditures and the usage of additional care were included, as these factors are closely related to health status and can be linked to the likelihood of using e-health interventions. Research indicates that individuals with greater health needs are more likely to use health information technology, and those with poorer health tend to search for health information online more frequently than their healthier peers [16, 53, 54]. While not direct indicators of health status, healthcare expenditures and the use of additional care are closely associated with individuals’ health status and care utilization patterns. Including these variables allows for a better understanding of potential differences between OncoAppstore users and the general population. The total healthcare expenditures of individuals were analysed, together with different categories of healthcare expenditures, including general practitioners’ care, hospital care, paramedical care, primary and specialist mental healthcare, and pharmaceutical expenditures. These expenses were calculated for the year prior to the year of diagnosis of an individual. For example, if an individual was diagnosed with cancer in 2021, the expenses for the year 2020 were calculated. However, this analysis could not be conducted for non-users diagnosed before 2018 or after 2022, as data on the year of diagnosis were unavailable beyond this point. As a result, comparisons between users and non-users were limited to those diagnosed between 2018 and 2022. The differences in healthcare expenditures between years were corrected for inflation based on Consumer Price Indexes[55].

In addition, it was analysed whether individuals received care under the Social Support Act, which is care provided by municipalities responsible for assisting people who cannot independently arrange the care and support they need [56]. The Social Support Act aims to help people live at home for as long as possible. Furthermore, we analysed whether individuals received care under the Long-Term Care Act [58]. The Long-Term Care Act regulates heavy, intensive care for vulnerable older adults, people with disabilities, and people with mental illness [57].

#### Proximity of care

Proximity of care was included in the analysis, as individuals in rural areas tend to use e-health less frequently, and healthcare proximity is closely linked to the rurality of their residence [30, 31]. To determine healthcare proximity, the distance between a person’s home address and the nearest general practitioner and hospital was measured. The average number of general practitioners and hospitals within a 5-km radius of their homes was also calculated.

Table 1 provides a summary of the study measures from the three different data sources.

**Table 1:** Summary of study measures

Source*	Category	Measures	Year of data included
IKNL	OncoAppstore (OS) usage	OS user	2023–2024
IKNL (NCR)	Clinical data of OS users	Year of diagnosis and cancer type	2018–2024
DHD	Clinical data of the general population of cancer survivors	Year of diagnosis, cancer type	2018–2022
SN	Sociodemographic characteristics	Gender, age, level of education, country of origin	n.a
SN	Income, benefits, and debts	Standardized household income, benefits, debts (i.e. restructuring and/or health insurance defaulters)	2023
SN	Healthcare expenditures	Total healthcare expenditures, expenditures for general practitioners’ (GP) care, hospital care, paramedical care, primary and specialist mental healthcare, and pharmacy	2017–2021 (year prior to year of diagnosis was used)
SN	Additional care reception	Care under the Social Support Act, Care under the Long-term Care Act	2023
SN	Proximity of care	Distance between home address and the nearest GP and hospital, number of GPs and hospitals within a 5-km radius of home	2022

*IKNL, The Netherlands Comprehensive Cancer Organization; *NCR*, Netherlands Cancer Registry; *DHD*, Dutch Hospital Data (DHD); S*N*, Statistics Netherlands

### Statistical analysis

First, all data were linked using R statistical software (version 4.2.3). Statistical analyses were then performed using SPSS (version 25) [58]. Descriptive analyses were performed to examine the distribution of variables for both OncoAppstore users and non-users. Statistical analyses were then conducted to determine whether any differences in the variables between the two groups, as outlined in Table 2, were statistically significant. Mann–Whitney *U* tests were conducted to assess the association between OncoAppstore usage and the continuous variables. Chi-square tests were conducted to investigate whether the categorical variables were differently distributed between OncoAppstore users and non-users. In one case (i.e. the ‘debts’ variable), where less than 80% of expected frequencies were greater than 5—thus violating an assumption of the chi-square test—a Fisher’s exact test was conducted. For the three variables that had three categories or more with significant differences between OncoAppstore users and non-users, additional tests were performed to assess in which of the categories the difference existed. This was done for the variables ‘educational level’, ‘income’, and ‘year of diagnosis’. To this end, a dummy variable was created for every category that indicated whether a person belonged to that particular category or not (e.g. highly educated or not). For every dummy variable, a chi-square test was conducted to test whether the dummy category was associated with OncoAppstore usage. This indicates whether, for example, the percentage of highly educated OncoAppstore users differs significantly from the percentage of highly educated non-users.

## Results

### Study characteristics

In total, 368 individuals who used the OncoAppstore gave permission for their data to be used anonymously for scientific research. Twenty-eight cases could not be linked, likely due to typographical errors in the linking information, such as date of birth and changes in postal code over the years. Additionally, 29 users were excluded for not meeting the inclusion criteria; they were either diagnosed before 2018 (*n* = 18) or had a cancer type outside the specified range of ICD-10 codes (*n* = 11). This resulted in 307 users who were compared to the 261.377 non-users that met the inclusion criteria. The most common cancer types were breast (53.8% in the user group and 21.6% in the non-user group), digestive system (10.8% and 20.6%), lymphoid hematopoietic and related tissue (7.5% and 8.4%), male genital tract (7.2% and 11.9%), female genital tract (6.6% and 6.1%), respiratory system and intrathoracic organs (5.2% and 7.2%), and kidney and urinary tract (3.3% and 9.1%). Of the 307 OncoAppstore users, 121 were diagnosed in 2023 and 46 in 2024. The distribution of diagnosis years from 2018 to 2022 is shown in Table 2, along with other user characteristics. Data for 2023 and 2024 are not included in the table, as no general population data were available for these years. The results of the statistical analyses are presented in Tables 2, 3, and 4 and further detailed below.

When considering sociodemographic and clinical variables (Table 2), OncoAppstore users were significantly younger compared to non-users (M = 56.5, SD = 10.5 vs. M = 68.3, SD = 13.3, *p* < 0.001). Additionally, gender was significantly associated with OncoAppstore usage (*p* < 0.001). The user group consisted of more females than non-users (79.2% vs. 54.0%). Also, there was a significant association between educational level and OncoAppstore usage (*p* < 0.001). There were significantly more individuals with a ‘High’ and significantly fewer individuals with a ‘Middle’ or ‘Low’ educational level in the user group compared to non-users (*p* < 0.001; *p* = 0.012; *p* < 0.001, respectively). There was also a significant influence of country of origin on OncoAppstore usage (*p* = 0.002), with users more frequently originating from the Netherlands (*p* < 0.001) and less frequently from outside Europe (*p* = 0.002) compared to non-users. Furthermore, the year of diagnosis was significantly associated with OncoAppstore usage (*p* < 0.001). OncoAppstore users were less often diagnosed in 2019 and 2020 and more often in 2022 than non-users (*p *= 0.033, *p *= 0.032, and *p* < 0.001, respectively).

**Table 2:** Study characteristics and results of the analyses of the sociodemographic variables and year of diagnosis (N=261.684)

	OS users (N=307)	Non-users (N=261.377)	p-value^1^	Missing OS users / non-users n (%)
**Age**	56.5 (10.5)	68.3 (13.3)	<.001***	0 (0%) / 0 (0%)
**Gender**			<.001***	0 (0%) / 0 (0%)
*Male *	64 (20.8%)	120,114 (46.0%)		
*Female*	243 (79.2%)	141,263 (54.0%)		
**Educational level **			<.001***	82 (26.7%) / 104.471 (40.0%)
*Low *	<10 (<4.4%)^2^	33,908 (30.3%)	<.001***	
*Middle *	>68 (>30.3%)^2^	43,495 (38.9%)	.012*	
*High *	147 (65.3%)	34,503 (30.8%)	<.001***	
**Country of origin**			.002**	0 (0%) / 0 (0%)
*The Netherlands*	280 (91.2%)	218,896 (83.7%)	<.001***	
*Europe (excl. the Netherlands)*	13 (4.2%)	17,022 (6.5%)	.106	
*Outside Europe*	14 (4.6%)	25,459 (9.7%)	.002**	
**Year of diagnosis**^3^			<.001***	0 (0.0%) / 0 (0%)
*2018*	18 (12.9%)	49,145 (18.8%)	.072	
*2019*	17 (12.1%)	50,268 (19.2%)	.033*	
*2020*	16 (11.4%)	48,295 (18.5%)	.032*	
*2021*	31 (22.1%)	55,159 (21.1%)	.763	
*2022*	58 (41.4%)	58,510 (22.4%)	<.001***	

Continuous variables presented as mean with SD, and categorical variables presented as counts with percentages. OS: OncoAppstore. Significance levels: *** = *p *< .001, ** = *p* < .01, * = *p *< .05. ^1^P-values in rows of category names indicate overall significance of the variable. P-values in rows of different categories indicate significance of a category compared to all other categories. P-values for categorical variables are based on Chi-square tests and Fisher’s exact tests (see Method, section Statistical analysis). P-values for continuous variables are based on Mann-Whitney-U tests. ^2^All values <10 and the corresponding percentages, together with the value and percentage of another category (i.e. ‘Middle’) are reported this way in accordance with SN guidelines to prevent disclosure risk. ^3^As there were no data available of non-users for 2023 and 2024, these years were excluded from the statistical analysis.

When considering income and benefits (Table 3), the distribution of income significantly differed between users and non-users (*p* < 0.001). Users had significantly more often a high income and significantly less often a low income compared to non-users (*p* < 0.001; *p* < 0.001, respectively). Furthermore, receiving benefits or having debts was not significantly associated with OncoAppstore usage.

**Table 3:** Study characteristics and results of the analyses of the income-related variables (N=261.684)

	OS users (N=307)	Non-users (N=261.377)	p-value^a^	Missing OS users / non-users n (%)
**Income**				
*Low *	45 (14.7%)	102.059 (39.8%)	<.001***	
*Middle *	132 (43.0%)	104.722 (40.9%)	.445	
*High*	130 (42.3%)	49.546 (19.3%)	<.001***	
**Benefits**^b^			.824	0 (0%); 2.016 (1.5%)
*No *	181 (65.0%)	84.406 (65.0%)		
*Yes*	100 (35.0%)	45.360 (35.0%)		
**Debts**			.547	0 (0.0%) / 0.0 (0.0%)
*No *	>297 (>96.7%)^c^	259,837 (99.4%)		
*Yes*	<10 (<3.3%)^c^	1540 (0.6%)		

Categorical variables presented as counts with percentages. *OS,* OncoAppstore. Significance levels: ***p < 0.001. ^a^*p*-values in rows of category names indicate overall significance of the variable. *p*-values in rows of different categories indicate significance of a category compared to all other categories. *p*-values for categorical variables are based on chi-square tests and Fisher’s exact tests. ^b^Benefits: unemployment, disability, social assistance, or other social security benefits. Only individuals aged ≤ 67 years were included in this analysis (OS users *n* = 281, non-users *n* = 131.782). ^c^Debts: Undergoing debt restructuring and/or being a health insurance defaulter. All values < 10 and the corresponding percentages, together with the value and percentage of another category (i.e. ‘No debts’) are reported this way in accordance with SN guidelines to prevent disclosure risk

Regarding healthcare expenditures (Table 4), total healthcare expenditures (*p* < 0.001), general practitioner expenditures (*p* < 0.001), hospital expenditures (*p* < 0.001), and pharmaceutical expenditures (*p* < 0.001) were significantly lower among users compared to non-users in the year before diagnosis. Primary and specialist mental healthcare expenditures were significantly higher (*p *= 0.007 and *p* = 0.004 resp.) among users compared to non-users. There were no significant differences in paramedical care expenditures. Receiving care under the Social Support Act and receiving care under the Long-term Care Act were negatively associated with OncoAppstore usage (*p *< 0.001; *p* = 0.001, respectively), suggesting that OncoAppstore users were less likely to use these additional forms of care. Regarding the proximity of healthcare facilities, the groups did not differ significantly in either the distance to the nearest GP and hospital or the number of hospitals and GPs within 5 km of their homes.

**Table 4:** Study characteristics and results of the analyses of the healthcare expenditures and usage, and proximity of care variables (N=261.684)

	OS users (N=307)	Non-users(N=261.377)	p-value^a^	Missing OS users / non-users (%)
Healthcare expenditures and usage				
Total healthcare expenditures	2559.3 (5350.7)	5235.6 (11295.9)	<.001***	46 (15.0%) / 2.058 (0.8%)
General practitioner (GP) care expenditures	171.9 (79.3)	214.8 (154.3)	<.001***	46 (15.0%) / 2.058 (0.8%)
Hospital care expenditures	1465.3 (3918.8)	3593.5 (9526.5)	<.001***	46 (15.0%) / 2.058 (0.8%)
Primary mental health care expenditures	46.8 (250.0)	11.8 (121.1)	.007**	167 (54.4%) / 2.058 (0.8%)^b^
Specialist mental health care expenditures	581.2 (3718.3)	158.2 (2290.5)	.004**	167 (54.4%) / 2.058 (0.8%)^b^
Paramedical care expenditures	47.3 (221.5)	54.6 (296.5)	.521	46 (15.0%) / 2.058 (0.8%)
Pharmaceutical expenditures	203.6 (442.5)	550.1 (2296.7)	<.001***	46 (15.0%) / 2.058 (0.8%)
**Care receival under the Social Support Act**			<.001***	0 (0.0%) / 0 (0%)
*No *	292 (95.1%)	221,683 (84.8%)		
*Yes*	15 (4.9%)	39,694 (15.2%)		
**Care receival under the Long-term Care Act**			.001**	0 (0.0%) / 0 (0%)
No	>297 (>96.7%)^c^	252.245 (96.5%)		
Yes	<10 (<3.3%)^c^	9132 (3.5%)		
**Proximity of care**				
Distance from home to the nearest hospital (in meters)	7250.1 (5040.7)	7623.6 (5850.1)	.780	16 (5.2%) / 24.425 (9.3%)
Distance from home to the nearest GP (in meters)	977.0 (711.7)	1070.5 (987.0)	.887	16 (5.2%) / 24.425 (9.3%)
Number of hospitals within five kilometers	0.58 (0.8)	0.56 (0.8)	.762	16 (5.2%) / 24.425 (9.3%)
Number of GPs within five kilometers	15.80 (20.8)	14.4 (17.9)	.561	16 (5.2%) / 24.425 (9.3%)

Continuous variables are presented as means with standard deviation, and categorical variables are presented as counts with percentages. *OS*, OncoAppstore. Significance levels: ***p < 0.001, **p < 0.01. ^a^*p*-value in rows of category names indicates overall significance of the variable. *p*-values in rows of different categories indicate significance of a category compared to all other categories. *p*-values for categorical variables are based on chi-square tests and Fisher’s exact tests. *p*-values for continuous variables are based on Mann–Whitney *U* tests. ^b^Data on primary and specialist mental healthcare expenditures were not yet available for 2022, explaining the high number of missing values among OS users (i.e. those diagnosed in 2023, for whom healthcare expenditures were calculated for 2022). ^c^All values < 10 and the corresponding percentages, together with the value and percentage of another category (i.e. ‘No receival’) are reported this way in accordance with SN guidelines to prevent disclosure risk

## Discussion

This study aimed to compare users of the OncoAppstore with the general population of cancer survivors diagnosed between 2018 and 2022 based on sociodemographic, clinical, income, and healthcare usage factors.

Regarding sociodemographic and income-related factors, OncoAppstore users tended to have higher educational levels and incomes, were more likely to be female, younger on average, and more often of Dutch origin compared to non-users. These findings indicate that the early uptake of the OncoAppstore is predominantly driven by individuals with higher socioeconomic status (SES), consistent with prior research. For instance, [58] demonstrated that individuals with higher SES are the first to adopt and benefit most from innovative technologies in health [59]. Other studies have shown that individuals with lower SES are often harder to reach for participation in digital interventions. In particular, digital self-management interventions are used less frequently by those with lower educational levels and adults over 65 years old [60, 61]. People with lower educational levels and older people often have less e-health literacy (i.e. the ability to use emerging information and communications technologies to improve or enable health and health care) [18]. Cancer survivors with lower digital literacy have less access to and tend to gain less from online resources than those with higher digital literacy [18, 62].These patterns suggest that OncoAppstore users are likely able to engage with the platform earlier because they possess the internal and external resources (e.g. educational level, digital literacy, income) that facilitate its proactive use.

Remarkably, a larger platform from a well-known source that cancer survivors frequently engage with, where costs are eliminated and awareness is widely spread, still seems to attract the same type of cancer survivors as observed in previous studies on e-health interventions. While the introduction of the credit and the centralized, reliable platform may have supported the use of digital self-management programs, they do not appear to have provided a strong incentive for potential lower-SES users to engage actively. Other factors—such as personal circumstances, health status, or digital literacy—may have played a more influential role in determining uptake. Moreover, it is important to clarify that the OncoAppstore is not meant to replace specific forms of care or reach all potential users. Its primary purpose is to enhance access to evidence-based self-management interventions for cancer survivors who may benefit from them. While the OncoAppstore currently appeals mainly to a specific group of patients, those who use it may reduce their reliance on formal healthcare services. This could potentially free up capacity among healthcare professionals, allowing them to focus more on individuals who are less able to address their challenges through digital self-management interventions. Whether this shift in healthcare utilization actually occurs should be explored further in future studies.

Our findings show that users have higher primary and specialist mental healthcare expenditures in the year before diagnosis compared to non-users, likely because many apps on the platform focus on mental well-being. This focus likely attracts individuals who were already seeking treatment for mental health-related issues. To fully interpret these and the other significant findings on healthcare expenditures, additional information is needed.

In contrast to other research showing that cancer survivors in rural areas use informational support applications less frequently, the current study found no association between OncoAppstore usage and the distance to healthcare services [21]. This suggests that in the compact geography of the Netherlands (i.e. a relatively small country with limited travel distances), OncoAppstore usage is not influenced by healthcare service proximity. This could be different for larger and more widely spread countries.

The current study has several strengths and limitations, which will be discussed below. An important strength of the study is the use of national registry data from Statistics Netherlands, NCR, and DHD. These data are often representative of the entire population, reducing selection bias. Additionally, the data are well maintained, ensuring high quality and accuracy. Furthermore, the OncoAppstore is incorporated into the well-known platform Kanker.nl, with a large reach among cancer survivors, that aims to promote the usage of online interventions. It provided a very interesting case to investigate the effects on usage among different types of cancer survivors.

There are also some limitations. First, there are some shortcomings in the data as mentioned in the Method section. A total of 368 individuals used the OncoAppstore and consented to the use of their data for scientific research. This number represents approximately 10% of the 3,620 OncoAppstore users (further detailed in the paper by de Korte et al.) [37]. However, some of these 3620 users are relatives, cancer survivors diagnosed before 2018, or individuals who only requested the health credit without purchasing an application and are therefore excluded from our target population. The relatively small sample size and the self-selection may have introduced bias, particularly if certain groups—such as women and individuals with higher education levels—were more likely to consent to data sharing. Furthermore, data on the year of diagnosis and cancer type were obtained differently for users and non-users. NCR data were used for users, while for the general population of cancer survivors (non-users), NCR data were unavailable to match a control group, so DHD data were used. Specifically, hospital admission records from 2018 to 2022 with a cancer diagnosis were utilized to identify cancer survivors in the general population and determine their year and type of diagnosis. However, some individuals may have been diagnosed with cancer but not admitted to the hospital during this period, for example, due to a wait-and-see approach to treatment. These factors could have impacted the validity of the data. Finally, the results were not adjusted for multiple testing as it concerned explorative research. Some *p*-values were close to the conventional threshold of 0.05, which calls for cautious interpretation.

Based on the findings of this study, several implications for policy and practice can be identified. It is important to recognize that some cancer survivors may face barriers to accessing digital self-management programs. Various strategies can be employed to engage a more diverse group of users, particularly those who are currently less engaged with the OncoAppstore. Active and tailored recruiting strategies, such as face-to-face or personal contact, can be effective as they help create a sense of security and increase engagement [28, 61, 63, 64]. Another promising strategy might be to use the social (i.e. relatives and healthcare professionals) and existing local networks (i.e. community centers) of people with lower SES [65, 66]. However, reaching individuals with lower SES remains challenging as there is little evidence for effective strategies to reach these individuals [63, 67]. Additionally, offering information and applications in multiple languages and providing content in various formats (audio, video, text) on the OncoAppstore could help increase usage among culturally and linguistically diverse populations [68–70]. Furthermore, ensuring that community sensitivities are considered and using culturally relevant examples could further enhance engagement [68, 71]. To attract individuals with limited digital literacy, it would be helpful to increase their digital literacy through (culturally tailored) educational programs, and the OncoAppstore interventions should be easily accessible and user-friendly [19].

Several directions for future research can be identified. Firstly, future research could follow up on the current study by including larger groups of OncoAppstore users, particularly those who used it after the data collection period of the current study to examine whether uptake patterns in this group are different from the group of early users. This will provide more comprehensive insights and determine if early users differ from later users of the OncoAppstore. Future research should also examine the effects of the OncoAppstore on cancer survivors’ (perceived) health outcomes, quality of life, healthcare utilization, work ability, and lifestyle factors. For example, longitudinal research could examine these factors before and after OncoAppstore usage. Additionally, further research, including qualitative studies, can be conducted to explore the perspectives and attitudes of underrepresented groups of cancer survivors towards the OncoAppstore. Also, future research can be conducted to explore how the examined characteristics interrelate and influence each other.

## Conclusion

This study is the first to use nationwide registry data to examine differences between users of a digital self-management intervention platform—one that offers high-quality interventions, provides a digital health credit, and comes from a well-known source—and the general population of cancer survivors (non-users). Our findings demonstrate that the OncoAppstore is currently used predominantly by highly educated, younger females with high incomes, originating in the Netherlands. Despite the platform’s large reach and the provision of a digital health credit, some groups remain underrepresented. Still, the OncoAppstore is not specifically designed to replace certain forms of care or reach all potential users. Its primary goal is to enhance and simplify access to evidence-based self-management interventions for cancer survivors. To promote broader inclusivity and extend the platform’s reach, targeted strategies could be implemented to engage a more diverse group of cancer survivors. Additionally, further research is needed to assess the OncoAppstore’s initial effects on cancer survivors’ health, quality of life, and healthcare utilization and to explore potential differences in uptake patterns between early and late adopters.

## Data Availability

The data generated and analysed during this study are available from the corresponding author upon reasonable request.

## References

[1] NKR (2024) Survival per year from diagnosis, Relative survival (in Dutch: Overleving per jaar vanaf diagnose, Relatieve overleving). https://nkr-cijfers.iknl.nl/viewer/relatieve-overleving-per-jaren-na-diagnose?language=nl_NL&viewerId=bd115fb0-d35c-4352-8426-7f805a506d0c. Accessed 03 Jun 2024

[2] Brandenbarg D, de Bock G, Berendsen A (2018) Follow-up and monitoring for cancer (in Dutch: Nazorg en controle voor kanker). Huisarts en wetenschap 61:18–22. 10.1007/s12445-018-0186-0

[3] Mayer DK, Nasso SF, Earp JA (2017) Defining cancer survivors, their needs, and perspectives on survivorship health care in the USA. Lancet Oncol 18(1):e11. 10.1016/S1470-2045(16)30573-3 28049573 10.1016/S1470-2045(16)30573-3

[4] Stubblefield MD (2017) The underutilization of rehabilitation to treat physical impairments in breast cancer survivors. PM R 9(9S2):S317–S323. 10.1016/j.pmrj.2017.05.010 28942906 10.1016/j.pmrj.2017.05.010

[5] Xu A, Wang Y, Wu X (2019) Effectiveness of e-health based self-management to improve cancer-related fatigue, self-efficacy and quality of life in cancer patients: systematic review and meta-analysis. J Adv Nurs 75:3434–3447. 10.1111/jan.14197 31566769 10.1111/jan.14197

[6] NFK (2021) Social support, understanding, and follow-up in cancer: what is your experience? (in Dutch: Sociale steun, begrip en nazorg bij kanker: wat is jouw ervaring?). https://doneerjeervaring.nl/media/1/Downloads/211025a-DJE-sociale-steun-zeldz2_rapportage_finaal.pdf. Accessed 21 Feb 2025

[7] van den Berg SW, Gielissen MF, Custers JA et al (2015) Breath: web-based self-management for psychological adjustment after primary breast cancer–results of a multicenter randomized controlled trial. J Clin Oncol. 10.1200/jco.2013.54.9386 26169621 10.1200/JCO.2013.54.9386

[8] Melissant HC, de Verdonck- Leeuw IM, Lissenberg-Witte BI et al (2018) ‘Oncokompas’, a web-based self-management application to support patient activation and optimal supportive care: a feasibility study among breast cancer survivors. Acta Oncol 57:924–934. 10.1080/0284186x.2018.1438654 29451059 10.1080/0284186X.2018.1438654

[9] Kanera IM, Bolman CA, Willems RA et al (2016) Lifestyle-related effects of the web-based Kanker Nazorg Wijzer (cancer aftercare guide) intervention for cancer survivors: a randomized controlled trial. J Cancer Surviv 10:883–897. 10.1007/s11764-016-0535-6 26984534 10.1007/s11764-016-0535-6 PMC5018034

[10] van Deursen L, Versluis A, van der Vaart R et al (2022) Ehealth interventions for Dutch cancer care: systematic review using the triple aim lens. JMIR Cancer 8:e37093. 10.2196/37093 35699991 10.2196/37093 PMC9240931

[11] Willems RA, Lechner L, Verboon P et al (2017) Working mechanisms of a web-based self-management intervention for cancer survivors: a randomised controlled trial. Psychol Health 32:605–625. 10.1080/08870446.2017.1293054 28276741 10.1080/08870446.2017.1293054

[12] Van Den Berg S, Gielissen M, Ottevanger N et al (2011) The BREATH intervention: online self-management to facilitate adjustment after curative breast cancer. Psychooncology 20:67–68. 10.1186/1471-2407-12-394

[13] Kim SH, Sung JH, Yoo S-H (2023) Effects of digital self-management symptom interventions on symptom outcomes in adult cancer patients: a systematic review and meta-analysis. Eur J Oncol Nurs 66:102404. 10.1016/j.ejon.2023.102404 37517339 10.1016/j.ejon.2023.102404

[14] Rakers MM, van Os HJ, Recourt K et al (2023) Perceived barriers and facilitators of structural reimbursement for remote patient monitoring, an exploratory qualitative study. Health Policy and Technology 12:100718. 10.1016/j.hlpt.2022.100718

[15] van Deursen L, van der Vaart R, Chavannes NH et al (2024) What is needed for improved uptake and adoption of digital aftercare programs by cancer survivors: a mixed methods study applying the COM-B model. J Cancer Surviv. 10.1007/s11764-024-01635-x 38965131 10.1007/s11764-024-01635-x PMC12906513

[16] Choi NG, DiNitto DM (2013) Internet use among older adults: association with health needs, psychological capital, and social capital. J Med Internet Res 15:e2333. 10.2196/jmir.2333 10.2196/jmir.2333 PMC3668603 23681083

[17] Jensen JD, King AJ, Davis LA et al (2010) Utilization of internet technology by low-income adults: the role of health literacy, health numeracy, and computer assistance. J Aging Health 22:804–826. 10.1177/0898264310366161 20495159 10.1177/0898264310366161

[18] Neter E, Brainin E (2012) Ehealth literacy: extending the digital divide to the realm of health information. J Med Internet Res 14:e19. 10.2196/jmir.1619 22357448 10.2196/jmir.1619 PMC3374546

[19] Meurk C, Leung J, Hall W (2016) Establishing and governing e-mental health care in Australia: a systematic review of challenges and a call for policy-focussed research. J Med Internet Res 18:e10. 10.2196/jmir.4827 26764181 10.2196/jmir.4827 PMC4730106

[20] Duplaga M, Turosz N (2022) User satisfaction and the readiness-to-use e-health applications in the future in Polish society in the early phase of the COVID-19 pandemic: a cross-sectional study. Int J Med Inform 168:104904. 10.1016/j.ijmedinf.2022.104904 36332522 10.1016/j.ijmedinf.2022.104904 PMC9595485

[21] Melhem SJ, Nabhani-Gebara S, Kayyali R (2023) Digital trends, digital literacy, and e-health engagement predictors of breast and colorectal cancer survivors: a population-based cross-sectional survey. Int J Environ Res Public Health 20:1472. 10.3390/ijerph20021472 36674237 10.3390/ijerph20021472 PMC9860554

[22] Bright MA, Fleisher L, Thomsen C (2005) Exploring e-health usage and interest among cancer information service users: the need for personalized interactions and multiple channels remains. J Health Commun 10:35–52. 10.1080/10810730500265609 16377599 10.1080/10810730500265609

[23] Van Dijk J (2020) The digital divide. John Wiley & Sons

[24] Coetzer JA, Loukili I, Goedhart NS (2024) The potential and paradoxes of ehealth research for digitally marginalised groups: a qualitative meta-review. Soc Sci Med 350:116895. 10.1016/j.socscimed.2024.116895 38710135 10.1016/j.socscimed.2024.116895

[25] Standaar L, van Tuyl L, Suijkerbuijk A (2025) Differences in eHealth access, use, and perceived benefit between different socioeconomic groups in the Dutch context: secondary cross-sectional study. JMIR Form Res 9:e49585. 10.2196/49585 39773883 10.2196/49585 PMC11751653

[26] Bidmon S, Terlutter R (2015) Gender differences in searching for health information on the internet and the virtual patient-physician relationship in Germany: exploratory results on how men and women differ and why. J Med Internet Res 17:e156. 10.2196/jmir.4127 26099325 10.2196/jmir.4127 PMC4526954

[27] Escoffery C (2018) Gender similarities and differences for e-health behaviors among US adults. Telemed e-Health 24:335–343. 10.1089/tmj.2017.0136 10.1089/tmj.2017.0136 28813630

[28] Reiners F, Sturm J, Bouw LJW et al (2019) Sociodemographic factors influencing the use of eHealth in people with chronic diseases. Int J Environ Res Public Health. 10.3390/ijerph16040645 30795623 10.3390/ijerph16040645 PMC6406337

[29] Hernandez LM (2009) Health literacy, eHealth, and communication: putting the consumer first: workshop summary. National Academies Press. 10.17226/12474 20662120

[30] Greenberg AJ, Haney D, Blake KD et al (2018) Differences in access to and use of electronic personal health information between rural and urban residents in the United States. J Rural Health 34(S1):s30–s38. 10.1111/jrh.12228 28075508 10.1111/jrh.12228 PMC5505819

[31] Wang J-Y, Bennett K, Probst J (2011) Subdividing the digital divide: differences in internet access and use among rural residents with medical limitations. J Med Internet Res 13:e25. 10.2196/jmir.1534 21371989 10.2196/jmir.1534 PMC3221347

[32] Vazquez CE, Mauldin RL, Mitchell DN (2024) Sociodemographic factors associated with using eHealth for information seeking in the United States: cross-sectional population-based study with 3 time points using health information national trends survey data. J Med Internet Res 26:e54745. 10.2196/54745 39141905 10.2196/54745 PMC11358649

[33] Kanker.nl (2023) Appstore. https://www.kanker.nl/hulp-en-ondersteuning/appstore?page=1. Accessed 11 Oct 2023

[34] IKNL (2023) Self-management for (former) cancer patients with the App Store on kanker.nl (in Dutch: Eigen regie voor (ex-)kankerpatiënten met Appstore op kanker.nl). https://iknl.nl/nieuws/2023/eigen-regie-voor-(ex-)kankerpatienten-met-appstore. Accessed 24 Feb 2025

[35] IKNL (2024) Self-management for (ex-)cancer patients with Appstore on kanker.nl (in Dutch: Eigen regie voor (ex-)kankerpatiënten met Appstore op kanker.nl). https://iknl.nl/nieuws/2023/eigen-regie-voor-(ex-)kankerpatienten-met-appstore. Accessed 03 Jun 2024

[36] IKNL (2023) About IKNL (in Dutch: Over IKNL). https://iknl.nl/over-iknl. Accessed 03 Jun 2024

[37] de Korte LvD AM, van der Lee ML, Aardoom JJ, Ezendam NPM, Heine P, Mols F, Mulder-Mertens H, Stouten MM, Lammens CRM (2024) Who uses self-management apps from the OncoAppstore: description of characteristics, symptoms, and functioning of a real-world user population. Manuscript submitted for publication10.1007/s11764-025-01855-940616696

[38] Kanker.nl (2023) About us (in Dutch: Over ons). https://www.kanker.nl/overig/over-ons. Accessed 24 May 2024

[39] KWF (2023) About dutch cancer society. https://www.kwf.nl/en/the-organization. Accessed 30 Mar 2023

[40] Kanker.nl (n.d.) Activate credit for the App Store (in Dutch: Tegoed activeren voor de Appstore). https://www.kanker.nl/hulp-en-ondersteuning/appstore/over-de-appstore. Accessed 01 Feb 2024

[41] GGD (2023) The test method: GGD AppStore methodology (in Dutch: De testmethode: GGD AppStore methodiek). https://www.ggdappstore.nl/Appstore/Testmethode. Accessed 11 Oct 2023

[42] de Korte LvD AM, van der Lee ML, Aardoom JJ, Heine P, Mulder-Mertens H, Stouten MM, Lammens CRM (2024) Implementation of the eHealth Appstore to improve access to self-management applications for Dutch people affected by cancer and their relatives: study protocol. Manuscript submitted for publication 10.1007/s00520-025-09720-2 PMC12228585 40616667

[43] Bakker BFM, van Rooijen J, van Toor L (2014) The system of social statistical datasets of Statistics Netherlands: an integral approach to the production of register-based social statistics. Stat J IAOS 30:411–424. 10.3233/SJI-140803

[44] Nictiz (2021) ICD-10 2021 v1a. https://terminologie.nictiz.nl/art-decor/claml?collection=icd10-nl-data. Accessed 18 Oct 2023

[45] IKNL (n.d.) NKR. https://iknl.nl/nkr. Accessed 13 Feb 2025

[46] DHD, 2023, Explore the possibilities of the LBZ (in Dutch: Ontdek de mogelijkheden van de LBZ), https://www.dhd.nl/producten-diensten/registratie-data/ontdek-de-mogelijkheden-van-de-lbz, 18–10–2023

[47] DHD (2023) About us (in Dutch: Over ons). https://www.dhd.nl/over-ons. Accessed 18 Oct 2023

[48] CBS (2023) Microdata: conducting your own research (in Dutch: Microdata: zelf onderzoek doen). https://www.cbs.nl/microdata. Accessed 19 Feb 2024

[49] Bakker BF, Van Rooijen J, Van Toor L (2014) The system of social statistical datasets of Statistics Netherlands: an integral approach to the production of register-based social statistics. Stat J IAOS: J Int Assoc Off Stat 30:411–424. 10.3233/SJI-140803

[50] CBS (2023) Educational level (in Dutch: Opleidingsniveau). https://www.cbs.nl/nl-nl/nieuws/2019/33/verschil-levensverwachting-hoog-en-laagopgeleid-groeit/opleidingsniveau. Accessed 11 Oct 2023

[51] CBS (2022) New classification of population by origin (in Dutch: Nieuwe indeling bevolking naar herkomst). https://www.cbs.nl/nl-nl/longread/statistische-trends/2022/nieuwe-indeling-bevolking-naar-herkomst/4-de-nieuwe-indeling-naar-geboren-in-nederland-en-herkomstland. Accessed 18 Oct 2023

[52] CBS, 2023, Natural Persons Debt Restructuring Act (in Dutch: Wet schuldsanering natuurlijke personen), https://www.cbs.nl/nl-nl/onze-diensten/maatwerk-en-microdata/microdata-zelf-onderzoek-doen/microdatabestanden/wsnppersbus-wet-schuldsanering-natuurlijke-personen, 03–06–2024

[53] Choi N (2011) Relationship between health service use and health information technology use among older adults: analysis of the US National Health Interview Survey. J Med Internet Res 13:e1753. 10.2196/jmir.1753 10.2196/jmir.1753 PMC3221375 21752784

[54] Flynn KE, Smith MA, Freese J (2006) When do older adults turn to the internet for health information? Findings from the Wisconsin longitudinal study. J Gen Intern Med 21(12):1295–1301. 10.1111/j.1525-1497.2006.00622.x 16995892 10.1111/j.1525-1497.2006.00622.x PMC1924748

[55] Statline (2023) Healthcare expenditure; volume and price developments (in Dutch: Zorguitgaven; volume- en prijsontwikkelingen). https://opendata.cbs.nl/#/CBS/nl/dataset/85103NED/table?dl=A402A. Accessed 30 Apr 2024

[56] Rijksoverheid (2023) Social support act (in Dutch: Wet maatschappelijke ondersteuning (Wmo)). https://www.rijksoverheid.nl/onderwerpen/zorg-en-ondersteuning-thuis/wmo-2015. Accessed 07 Apr 2023

[57] Rijksoverheid (2023) Long-term care act (in Dutch: Wet langdurige zorg (Wlz)). https://www.rijksoverheid.nl/onderwerpen/verpleeghuizen-en-zorginstellingen/wet-langdurige-zorg-wlz. Accessed 07 Apr 2023

[58] Team RDC (2007) R: A language and environment for statistical computing. https://www.r-project.org/. Accessed 19 Oct 2023

[59] Weiss D, Rydland HT, Øversveen E (2018) Innovative technologies and social inequalities in health: a scoping review of the literature. PLoS ONE 13:e0195447. 10.1371/journal.pone.0195447 29614114 10.1371/journal.pone.0195447 PMC5882163

[60] Keij B, Versluis A, Alblas E et al (2024) E-health monitor 2023. State of affairs in digital care (in Dutch: E-healthmonitor 2023. Stand van zaken digitale zorg). 10.21945/RIVM-2024-0008

[61] Al-Dhahir I, Reijnders T, Faber JS (2022) The barriers and facilitators of eHealth-based lifestyle intervention programs for people with a low socioeconomic status: scoping review. J Med Internet Res 24:e34229. 10.2196/34229 36001380 10.2196/34229 PMC9453585

[62] Verma R, Saldanha C, Ellis U (2022) Ehealth literacy among older adults living with cancer and their caregivers: a scoping review. J Geriatr Oncol 13:555–562. 10.1016/j.jgo.2021.11.008 34810146 10.1016/j.jgo.2021.11.008

[63] Stanczyk NE, Crutzen R, Bolman C et al (2013) Influence of delivery strategy on message-processing mechanisms and future adherence to a Dutch computer-tailored smoking cessation intervention. J Med Internet Res 15:e28. 10.2196/jmir.2153 23388554 10.2196/jmir.2153 PMC3636289

[64] Stuber JM, Middel CN, Mackenbach JD et al (2020) Successfully recruiting adults with a low socioeconomic position into community-based lifestyle programs: a qualitative study on expert opinions. Int J Environ Res Public Health 17:2764. 10.3390/ijerph17082764 32316344 10.3390/ijerph17082764 PMC7215437

[65] van Dongen JM, van Poppel MN, Milder IE et al (2012) Exploring the reach and program use of hello world, an email-based health promotion program for pregnant women in the Netherlands. BMC Res Notes 5:1–12. 10.1186/1756-0500-5-514 22999052 10.1186/1756-0500-5-514 PMC3500255

[66] Faber JS, Al-Dhahir I, Reijnders T (2021) Attitudes toward health, healthcare, and ehealth of people with a low socioeconomic status: a community-based participatory approach. Front Digit Health. 10.3389/fdgth.2021.690182 34713165 10.3389/fdgth.2021.690182 PMC8521920

[67] Bonevski B, Randell M, Paul C et al (2014) Reaching the hard-to-reach: a systematic review of strategies for improving health and medical research with socially disadvantaged groups. BMC Med Res Methodol 14:1–29. 10.1186/1471-2288-14-42 24669751 10.1186/1471-2288-14-42 PMC3974746

[68] Whitehead L, Talevski J, Fatehi F et al (2023) Barriers to and facilitators of digital health among culturally and linguistically diverse populations: qualitative systematic review. J Med Internet Res 25:e42719. 10.2196/42719 36853742 10.2196/42719 PMC10015358

[69] Sibuyi I-N, de la Harpe R, Nyasulu P (2022) A stakeholder-centered mHealth implementation inquiry within the Digital Health Innovation Ecosystem in South Africa: MomConnect as a demonstration case. JMIR Mhealth Uhealth 10:e18188. 10.2196/18188 35708756 10.2196/18188 PMC9247812

[70] O’Connor S, Hanlon P, O’Donnell CA et al (2016) Understanding factors affecting patient and public engagement and recruitment to digital health interventions: a systematic review of qualitative studies. BMC Med Inform Decis Mak 16:120. 10.1186/s12911-016-0359-3 27630020 10.1186/s12911-016-0359-3 PMC5024516

[71] Arsenijevic J, Tummers L, Bosma N (2020) Adherence to electronic health tools among vulnerable groups: systematic literature review and meta-analysis. J Med Internet Res 22:e11613. 10.2196/11613 32027311 10.2196/11613 PMC7055852

